# Multiple-cohort study of the elderly to determine the immunological characteristics and pathogenic mechanisms of severe community-acquired pneumonia caused by the low-virulence virus SARS-CoV-2 Omicron variant

**DOI:** 10.1038/s41421-023-00626-z

**Published:** 2023-12-05

**Authors:** Tianyu Lu, Qiuhong Man, Shuai Xia, Xiaohang Liu, Yan Yan, Xueying Yu, Yan Fu, Wanli Liu, Lu Lu, Shibo Jiang, Lize Xiong

**Affiliations:** 1 Key Laboratory of Medical Molecular Virology (MOE/NHC/CAMS), Shanghai Institute of Infectious Disease and Biosecurity, School of Basic Medical Sciences and Huashan Hospital, Shanghai Frontiers Science Center of Pathogenic Microorganisms and Infection, Fudan University, Shanghai, China; 2 Department of Laboratory Medicine, Shanghai Fourth People’s Hospital, School of Medicine, Tongji University, Shanghai, China; 3 State Key Laboratory of Membrane Biology, School of Life Sciences, Tsinghua-Peking Center for Life Sciences, Institute for Immunology, Beijing Advanced Innovation Center for Structural Biology, Beijing Key Lab for Immunological Research on Chronic Diseases, Beijing, China; 4 Shanghai Key Laboratory of Anesthesiology and Brain Functional Modulation, Clinical Research Center for Anesthesiology and Perioperative Medicine, Translational Research Institute of Brain and Brain-Like Intelligence, Shanghai Fourth People’s Hospital, School of Medicine, Tongji University, Shanghai, China

**Keywords:** Immunology, Cell biology

Dear Editor,

Low-virulence respiratory viruses can cause severe community-acquired pneumonia (Sev-CAP) in the elderly whose susceptibility is correlated with age-related factors as immunosenescence and complicated comorbidities. Its occurrence, hospitalization, severity, and mortality are higher in older adults and increase with aging^1^. The currently prevailing SARS-CoV-2 Omicron variant is a classic example of a low-virulence virus causing CAP in the elderly, while the young escape with mild or no symptoms^2,3^. Omicron variant infection in older adults leads to Sev-CAP with subsequent respiratory distress, lung injury, sepsis, and even multiorgan dysfunction^4^. Nevertheless, both immunological characteristics and pathogenic mechanisms of Sev-CAP induced by the low-virulence Omicron variant in older adults remain to be fully elucidated. Here, we investigated this issue in a multi-cohort study of elderly subjects infected by the Omicron variant via multiple immunological detections and single-cell RNA sequencing (scRNA-seq). This study was approved by the Ethics Committee of Shanghai Fourth People’s Hospital (No. 2022098-001 and 2022185-001).

Immunological features were primarily explored in Cohort-I (Fig. 1a), consisting of older adults belonging to a moderate group (MG, without CAP) and a severe group (SG, with CAP). Compared to MG, neutrophils, IL-6, and immunoglobulins were significantly higher in SG, whereas lymphocytes, monocytes, and TNF-α were in a much lower level, globally indicating elevated inflammatory and antibody responses but diminished antiviral immunity in Sev-CAP (Fig. 1b). Negative Spearman correlations were found between inflammatory indicators (neutrophils and IL-6) and antiviral indicators (lymphocytes, monocytes, and TNF-α) and between immunoglobulins and antiviral indicators, while positive correlations were found between inflammatory indicators, between antiviral indicators, and between neutrophils and immunoglobulins, suggesting their mutuality and influences on Sev-CAP development (Supplementary Fig. S1a). These factors were stratified by groups and compared between patients < 80 years and ≥ 80 years. Neutrophils, IL-6, and immunoglobulins seemed to be elevated in patients ≥ 80 years in both MG and SG, but antiviral indicators, such as lymphocytes (in MG and SG) and TNF-α (in MG), seemed to be diminished in patients ≥ 80 years, suggesting important roles of immunosenescence in Sev-CAP development (Supplementary Fig. S1b).

**Fig. 1:**
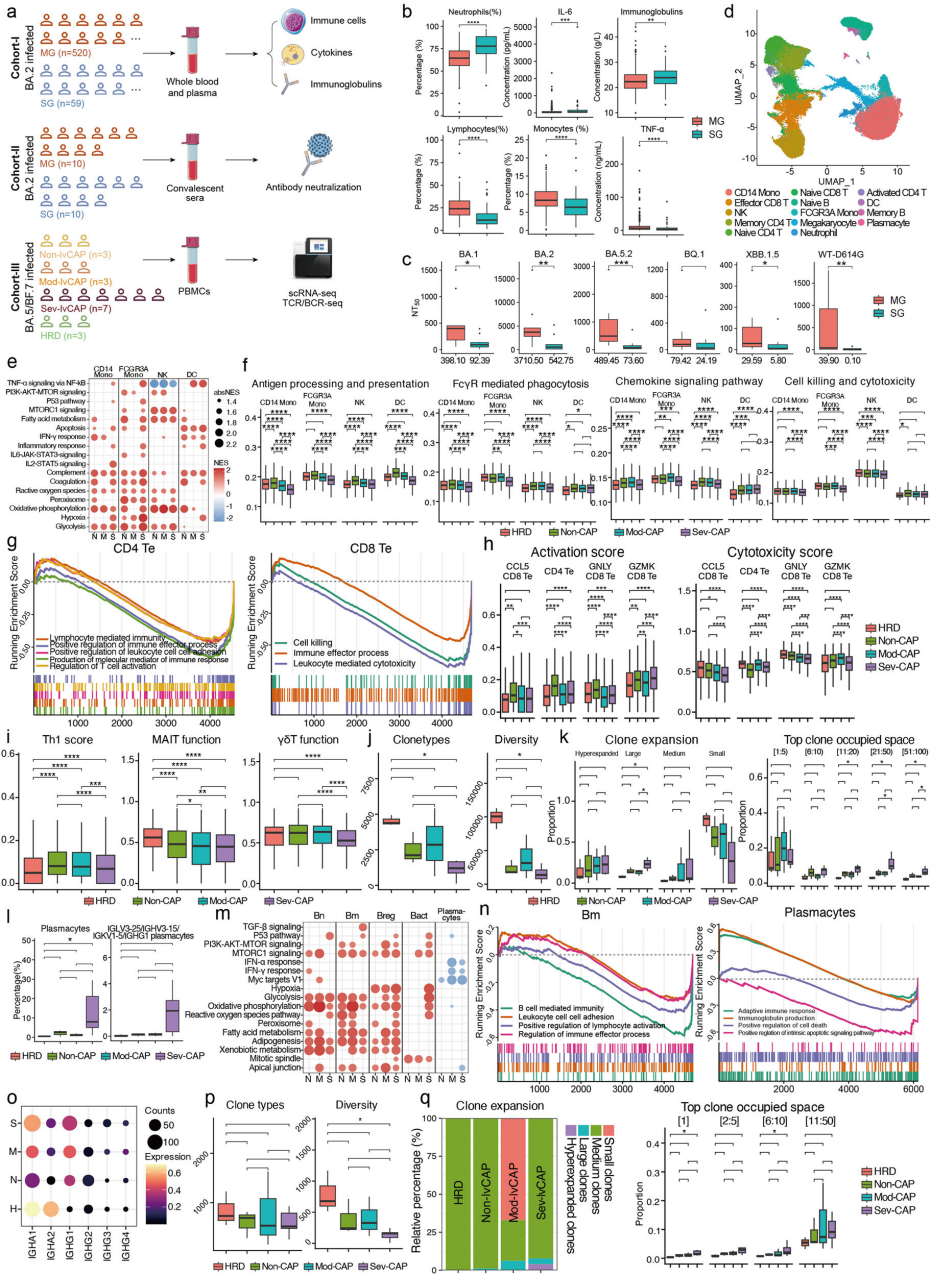
Immunological characteristics and pathogenic mechanisms of elderly patients infected by the Omicron variant. **a** Flow diagrams describing the study design. **b** Levels of immune indexes in MG (the moderate group) and SG (the severe group) of Cohort-I. **c** NT_50_ values in MG and SG of Cohort-II. Median NT_50_ values of each group were labeled at the bottom. **d** The Uniform Manifold Approximation and Projection (UMAP) display of all cell types in PBMCs of Cohort-III. Mono: monocyte; DC: dendritic cell; NK: natural killer cell. **e**, **m** GSEA results of Non-CAP (N), Mod-CAP (M), and Sev-CAP (S) versus HRD (H) in the innate immune cells (**e**) and B cells (**m**). Significant pathways were listed left. Dot colors represented Normalized Enrichment Scores (NESs) of these pathways, and dot sizes represented absolute NESs (absNESs). Red: upregulated; blue: downregulated. Bn: naive B; Bm: memory B; Breg: regulatory B; Bact: activated B. **f**, **h**, **i** Scores of pathway activities or cellular functions in different groups in the innate immune cells (**f**) and T cells (**h**, **i**). Te: effector T; MAIT: mucosal-associated invariant T; γδT: gamma-delta T. **g**, **n** Relevant GSEA results of Sev-CAP vs Non-CAP in T cells (**g**) and B cells (**n**). Relevant pathways were labeled and displayed as curves. *x* axis represented differential gene orders, and *y* axis represented running enrichment scores of these pathways. Vertical lines at the bottom represented enriched genes in these pathways. **j**, **k**, **p**, **q** Numbers of clone types, clone type diversity, situation of clone expansion, and top clone proportions in different groups in TCR (**j**, **k**) and BCR (**p**, **q**) repertoires. Types of clones with different expansion and ranks of top clones were labeled under the plot titles. **l** Plasmacyte proportions in different groups. **o** Expression of IGHA and IGHG genes in plasmacytes in different groups. Wilcoxon test was used for statistical analysis. *P value ≤ 0.05; **P value ≤ 0.01; ***P value ≤ 0.001; ****P value ≤ 0.0001; blank: *P* value > 0.05.

We further evaluated antibody neutralization in Cohort-II, consisting of MG and SG (Fig. 1a). Compared to MG, SG displayed the lower 50% neutralizing titer (NT_50_) against several Omicron subvariants and the ancestral strain, including BA.1 (0.23-fold), BA.2 (0.15-fold), BA.5.2 (0.15-fold), XBB.1.5 (0.20-fold), and WT-D614G (< 0.01-fold) (Fig. 1c). Therefore, despite elevated antibody responses, Sev-CAP patients showed weak antibody neutralization. Stratified by groups, NT_50_ value of patients ≥ 80 years was lower than that of patients < 80 years in all the Omicron subvariants in SG, although *P* values were not significant, suggesting possible correlations between aging and antibody neutralization in severe patients (Supplementary Fig. S1c).

To deeply investigate the immunological mechanisms, scRNA-seq and TCR/BCR-seq were performed in PBMCs of Cohort-III, consisting of Non-CAP (without CAP), Mod-CAP (moderate CAP), Sev-CAP (severe CAP), and HRD (healthy recovered donors) groups (Fig. 1a). Cell types in PBMCs were identified based on the expression of cell type-specific genes (Fig. 1d; Supplementary Figs. S2 and S3).

Innate immune cells were first studied. To discover global transcriptional characteristics, we performed Gene Set Enrichment Analysis (GSEA) between HRD and the other groups. Sev-CAP displayed upregulated pathway activities in hypoxia, inflammation, and apoptosis in multiple cells, while upregulation of some protective and functional pathways was absent or diminished (Fig. 1e). To discover factors determining CAP severity, we identified common differentially expressed genes (DEGs) in Mod-CAP and Sev-CAP (vs Non-CAP) and performed gene ontology (GO) enrichment, followed by GSEA between Sev-CAP and Non-CAP (Supplementary Fig. S4a–c). In CD14^+^ monocytes (CD14 Mono), pathways involving leukocyte activation and migration, leukocyte-mediated immunity, antigen processing and presentation, inflammatory responses, and cytokine production were enriched. According to the GSEA results, except viral genome replication, pathways and genes involving antigen processing and presentation and monocyte differentiation were downregulated in Sev-CAP. In natural killer (NK) cells, the cellular killing, migration, activation, response to virus, and regulation of inflammation were enriched. According to the results of GSEA and gene expression, cell killing/cytotoxicity, oxidative phosphorylation, and type-II IFN production were downregulated in Sev-CAP. In dendritic cells (DCs), except elevated inflammation, several pathways were downregulated in Sev-CAP, including antigen processing and presentation, IFN-α production, and oxidative phosphorylation. According to results of AUCell that calculates pathway activities (Fig. 1f), the activities of antigen processing and presentation and FcγR-mediated phagocytosis decreased with the increasing of disease severity in CD14 Mono, FCGR3A Mono, or DC. The activities of chemokine signaling and cell killing were also downregulated in Sev-CAP in their responsible cells. Based on the results above, impairment of antigen processing, cell killing, and phagocytosis, accompanied by hyperinflammation and energy deprivation, comprised the innate immunity hallmarks of Sev-CAP.

T cells were further classified into 18 subtypes (Supplementary Figs. S5a, b and S6). Pathways affecting T cell activation and functions, like IFN responses, IL-6-JAK-STAT3, and IL-2-STAT5, were limited in Sev-CAP in some cell types, whereas hypoxia was upregulated (Supplementary Fig. S5c). GO enrichment and GSEA were performed to further understand functional differences of effector T cells (Te). In CD4 Te, T cell-mediated immune effector process, activation, and adhesion were downregulated in Sev-CAP, along with genes involved in these pathways (Fig. 1g; Supplementary Fig. S5d). In integrated CD8 Te, cell killing, IFN-γ production, viral process, migration, and adhesion were enriched, and pathway and genes of cell killing/cytotoxicity and immune effector process were all downregulated in Sev-CAP (Fig. 1g; Supplementary Fig. S5e). Except in GZMK^+^ CD8 Te, Mod/Sev-CAP had lower activation scores compared with Non-CAP, and cytotoxicity scores were lower in Sev-CAP in CD8 Te (Fig. 1h). Moreover, Th1 differentiation and both mucosal-associated invariant T (MAIT) and γδT functions were restricted in Sev-CAP (Fig. 1i). These results revealed impairment of some specific functions of T cells in Sev-CAP. TCR repertoire of Sev-CAP seemed to have the fewest clone types and the least diversity, along with the increasing large clone proportion and proportions of top clones with the increase of CAP severity (Fig. 1j, k).

To study humoral immunity, B cells were further classified into 8 subtypes (Supplementary Figs. S7a–c and S8). First, Sev-CAP had a higher proportion of plasmacytes than other groups (Fig. 1l). GSEA results displayed elevated hypoxia in Sev-CAP in some cell types, also implying energy deprivation in Sev-CAP (Fig. 1m). GSEA was also performed between Sev-CAP and Non-CAP. In memory B cells (Bm), B cell immunity, adhesion, and activation were commonly downregulated in Sev-CAP, suggesting less functional Bm (Fig. 1n; Supplementary Fig. S7d). Moreover, their plasmacytes exhibited enhanced activities of immunoglobulin production and immune responses (Fig. 1n; Supplementary Fig. S7e). Genes of high-affinity antibodies, IGHA1 and IGHG1, showed increasing expression from Non-CAP to Sev-CAP (Fig. 1o). These results, together with the elevated plasmacyte proportion and Cohort-I results, implicated the increased proportion and excess functions of plasmacytes in Sev-CAP. Although clone types of BCR repertoire exhibited subtle differences, Sev-CAP seemed to have the lower BCR diversity and higher proportion of large and hyperexpanded clones than other groups, along with increasing top clone proportions with the increase of CAP severity (Fig. 1p, q).

Our results showed that Sev-CAP had impaired functions of innate immune cells and effector T cells, many of which are age-dependent and potently contribute to older adults’ susceptibility^5,6^. Therefore, these patients could not effectively initiate immunity to suppress the Omicron variant, which was termed as the delayed response by a previous study, resulting in exacerbated inflammation and lung pathology^7^. Next, the Sev-CAP exhibited a higher level of plasmacytes with immunoglobulin hyperproduction, but defective Omicron variant-specific neutralization, possibly attributed to age-related immune repertoire magnitude and inflammation as reported^6,8,9^. Finally, energy deprivation, which can affect immune cell functions and cytokine production, was remarkable in Sev-CAP. Therefore, although the Omicron variant is a low-virulence virus, it may still bring severe outcomes to older adults, considering their retarded oxidative phosphorylation uncovered before^10^. Although the high-virulence ancestral strain and the low-virulence Omicron variant have some similar characteristics, features of severe disease caused by the Omicron variant revealed by our study are still different from those caused by the ancestral strain. Severe patients infected by the ancestral strain were reported to have adaptive and hyperactivated NK, reduced CD16^+^ monocyte abundance, insufficient expression of pro-inflammatory cytokines in peripheral monocytes and lymphocytes, and elevated IFN-α responses in multiple T cell subtypes^11–14^, which were differential or uncharacteristic in our study.

### Supplementary information

Supplementary Information

## Data Availability

Data are available from the corresponding authors upon reasonable request.
