# Supplementary material for: Multiple-cohort study of the elderly to determine the immunological characteristics and pathogenic mechanisms of severe community-acquired pneumonia caused by the low-virulence virus SARS-CoV-2 Omicron variant
Source: Cell Discov. 2023 Dec 5;9:121. doi: 10.1038/s41421-023-00626-z (PMC10698024; doi:10.1038/s41421-023-00626-z)
Supplement: Supplementary file 1 — Supplementary Information [file 41421_2023_626_MOESM1_ESM.pdf]

**Supplementary Information For**

# Multiple-cohort study of the elderly to determine the immunological characteristics and pathogenic mechanisms of severe community-acquired pneumonia caused by the low-virulence virus SARS-CoV-2 Omicron variant

Tianyu Lu[1†], Qiuhong Man[2†], Shuai Xia[1†], Xiaohang Liu[3], Yan Yan[1], Xueying Yu[2], Yan Fu[2], Wanli Liu[3], Lu Lu[1*], Shibo Jiang[1*], Lize Xiong[4*]

[1] Key Laboratory of Medical Molecular Virology (MOE/NHC/CAMS), Shanghai Institute of Infectious Disease and Biosecurity, School of Basic Medical Sciences and Huashan Hospital, Shanghai Frontiers Science Center of Pathogenic Microorganisms and Infection, Fudan University, Shanghai 200030, China.

[2] Department of Laboratory Medicine, Shanghai Fourth People's Hospital, School of Medicine, Tongji University, Shanghai 200434, China.

[3] State Key Laboratory of Membrane Biology, School of Life Sciences, Tsinghua-Peking Center for Life Sciences, Institute for Immunology, Beijing Advanced Innovation Center for Structural Biology, Beijing Key Lab for Immunological Research on Chronic Diseases, Beijing, 100084, China.

[4] Shanghai Key Laboratory of Anesthesiology and Brain Functional Modulation, Clinical Research Center for Anesthesiology and Perioperative Medicine, Translational Research Institute of Brain and Brain-Like Intelligence, Shanghai Fourth People's Hospital, School of Medicine, Tongji University, Shanghai 200434, China.

† The authors contributed to the article equally.

* Co-corresponding authors: Lu Lu (lul@fudan.edu.cn), Shibo Jiang (shibojiang@fudan.edu.cn), and Lize Xiong (lizexiong@tongji.edu.cn).

**1. Supplementary Methods**

Study and cohort design

Detection of immune cells, cytokines, and immunoglobulin

Convalescent sera neutralization assay against SARS-CoV-2

scRNA-seq in PBMCs and data processing

Separation of PBMCs

Single-cell RNA sequencing

DEGs analysis and pathway enrichment

Gene or pathway activity scoring

TCR/BCR repertoire sequencing and data processing

**2. Supplementary Figures**

Supplementary Fig. S1

Supplementary Fig. S2

Supplementary Fig. S3

Supplementary Fig. S4

Supplementary Fig. S5

Supplementary Fig. S6

Supplementary Fig. S7

Supplementary Fig. S8

**3. Supplementary Tables**

Supplementary Table S1

Supplementary Table S2

Supplementary Table S3

Supplementary Table S4

Supplementary Table S5

**4. References**

## 1. Supplementary Methods

### *Study and cohort design*

This study was carried out between 2022 and 2023 in Shanghai Fourth People's Hospital and was approved by the ethics committee of Shanghai Fourth People's Hospital (No. 2022098-001 and 2022185-001). Omicron variant infection in patients was confirmed by real-time polymerase chain reaction. According to the Diagnosis and Treatment Scheme of Pneumonia Caused by Novel Coronavirus of China, patients were routinely diagnosed and treated, and disease severity was judged as four types: light, middle, heavy, and critically ill. Community-acquired pneumonia (CAP) existed in the middle type and severer, which was confirmed by clinical and/or radiological manifestations. The light type only showed mild upper-respiratory infection. Three cohorts were included in our study. Cohort-I/II contained 2 groups: MG (light, without CAP) and SG (severer types, with CAP). Cohort-III contained 4 groups: Non-CAP (light, without CAP), Mod-CAP (middle, moderate CAP), Sev-CAP (heavy and critically ill, severe CAP), and HRD (healthy recovered donors). Samples of Cohort-I and Cohort-III (except HRD) were collected from currently infected patients, and in Cohort-II and Cohort-III (HRD), samples were collected from recovered patients. Samples of Cohort-II were collected 2-5 days after recovery, and HRD samples of Cohort-III were collected $\geq$ 2 weeks after recovery. Detailed clinical characteristics were collected and compared to check patient heterogeneity (Supplementary Table S1-3).

***Detection of immune cells, cytokines, and immunoglobulin***

Whole blood and EDTA-anticoagulated plasma were collected from patients in Cohort-I for detection of immune cells and cytokines. Numbers and percentages of immune cells (leukocytes, neutrophils, lymphocytes, monocytes, eosinophils, and basophils) were tested in whole blood (BC-75000 Fully Automated Hematology Analyzer, Mindray, China). Cytokines (IL-6, IL-17A, IL-19, IFN-γ, TNF-α, IL-2, IL-1B, IL-5, IL-12, IL-8, and IL-4) were detected in EDTA-anticoagulated plasma by flow cytometry (DxFLEX, Beckman, USA). Immunoglobulin was calculated as total protein (detected by the Biuret method) minus albumin (detected by the bromocresol green colorimetric method) in EDTA-anticoagulated plasma.

***Convalescent sera neutralization assay against SARS-CoV-2***

Convalescent sera were collected from patients in Cohort-II, and neutralization assay was performed as previously described [1]. SARS-CoV-2 pseudotyped viruses (PsVs) BA.1, BA.2, BA.5.2, BQ.1, XBB.1.5, and WT-D614G were packaged in HEK-293T cells using their envelope plasmids and the backbone plasmid HIV-1 pNL4-3.Luc.R-E-. Caco-2 cells were seeded into wells of 96-well plates ($1\times10^4$ cells/well) 48-60 h before the assay. PsVs with or without serially diluted sera were added into cells for 12 h incubation, and the sera were 4-fold diluted with the maximum dilution of 1:10. Then, the culture medium was refreshed for another 48 h incubation.

Afterwards, cells were lysed, and luciferase values were measured by Luciferase Assay System (Promega, USA) to calculate the 50% neutralizing titer ($NT_{50}$) values.

***scRNA-seq in PBMCs and data processing***

Whole blood (2 mL) was collected from patients in Cohort-III and placed into EDTA anti-coagulated tubes. Samples were centrifugated at 500×g for 5 min at 4 °C, and the supernatant was removed with replenishment of the same volume of PBS. PBMCs were separated, and scRNA-seq was performed as described below. Feature-barcode matrices were read and merged into the scRNA-Seq object using Seurat (4.3.0) [2]. Then, the object was inspected qualitatively, and cells with 500~2500 gene numbers and < 5% mitochondrial genes were retained. *FindVariableFeatures, ScaleData,* and *RunPCA* were used to find hypervariable genes, normalize, and reduce dimensions. Then, batch effects were removed by Harmony (0.1.1), and the object was separated into several clusters via *FindNeighbors*, *FindClusters*, and *clustree*. Cluster-specific genes were calculated by *FindAllMarkers* to facilitate cell type identification. Afterwards, types were annotated based on expression of cell type-specific genes in each cluster (Supplementary Table S4).

***Separation of PBMCs***

The same volume of Ficoll-Paque PLUS (Cytiva, USA) was added to the bottom of EDTA anti-coagulated tubes, and tubes were centrifugated at 800×g for 20 min at 4 °C. After centrifugation, the PBMC layer in the middle was collected, and 10 mL of

DBPS were added for another centrifugation at 800×g for 5 min at 4 °C. Afterwards, the supernatant was aspirated off, and 1 mL of Red Blood Cell Lysis Solution (1×) (Miltenyi Biotec, USA) was added for erythrocyte lysis (3 min on ice). Lysis was terminated with 5 mL of DMEM medium (meilunbio, China) with 10% Fetal Bovine Serum (FBS) (Yeasen, China). PBMCs were centrifugated at 500×g for 10 min at 4 °C and washed with DMEM medium with 10% FBS for further use. Cell viability of each sample was examined, and samples with viability over 80% were eligible. Then, eligible samples were resuspended with DMEM medium with 10% FBS to the concentration of 700-1200 cells/μL.

***Single-cell RNA sequencing***

For single-cell preparation, Gel Beads in Emulsion (GEMs) were formed on 10x Genomics Chromium$^{TM}$ using Chromium Next GEM Single Cell 5' Kit (10x Genomics, USA), and subsequent reverse transcription was performed on Thermal Cycler (Bio-Rad, USA) for labeling. The cDNA was captured, amplified, and inspected according to the protocol. Then, the 5' gene library was established using 5'Library Kits (10x Genomics, USA) and quantitatively examined for final sequencing on Illumina Novaseq6000 (Illumina, USA). Finally, Cell Ranger (7.1.0) (10x Genomics, USA) was used for human genome mapping, quality control, and production of read counts.

***DEGs analysis and pathway enrichment***

Differences of gene expression among groups were calculated by *FindMarkers*. DEGs were determined with adjusted *P*-values < 0.05 and log-fold changes (logFCs) > 0.1. Gene Ontology (GO) enrichment was performed using the determined DEGs by clusterProfiler (4.6.2) [3]. The gene order calculated by *FindMarkers* was used to perform Gene Set Enrichment Analysis (GSEA) using gene sets of hallmark and GO biological process [4].

***Gene or pathway activity scoring***

Scores of gene or pathway activities were calculated by *AddModuleScore_UCell* of Seurat. Pathway genes used for scoring were listed in Supplementary Table S5.

***TCR/BCR repertoire sequencing and data processing***

Preparation of cell samples, formation of GEMs, and amplification and enrichment were similar to scRNA-seq described above using Chromium Next GEM Chip G Single Cell Kit (10x Genomics, USA) and Chromium Single Cell V(D)J Enrichment Kit (Human B Cell and T Cell) (10x Genomics, USA). Then, the library was constructed using 5'Library Kits for final sequencing on Illumina Novaseq6000. Finally, Cell Ranger (7.1.0) was used for calculating clonotypes and CDR3 sequences. Clone types, diversity, and proportions of top clones were evaluated by *repExplore*, *repDiversity*, and *repClonality* of immunarch (0.9.0) [5]. Situation of clone expansion was calculated by *repClonality* and scRepertoire (1.8.0) [6].

## 2. Supplementary Figures

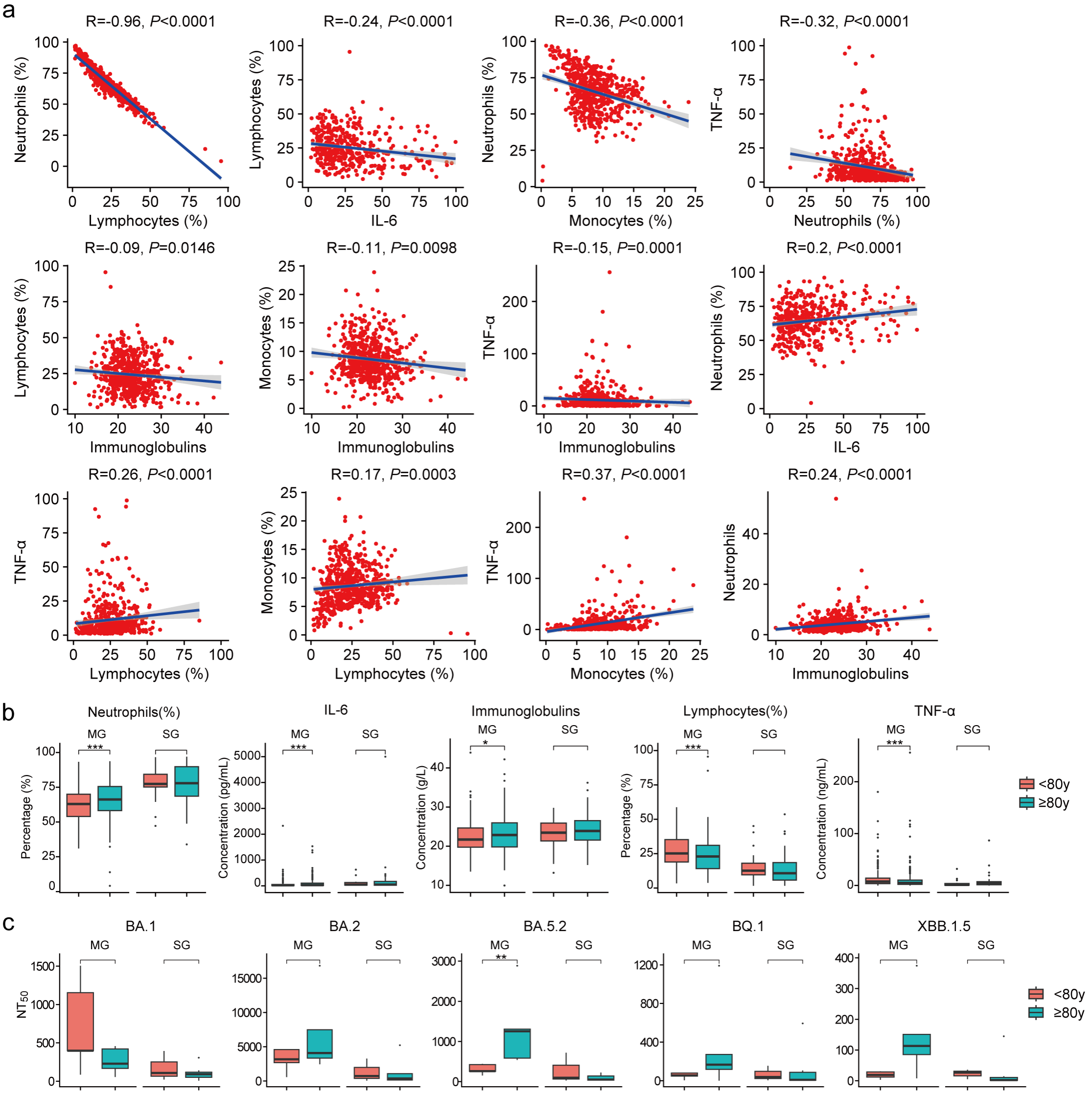


**Supplementary Fig. S1. Immunological characteristics of patients in Cohort-I and Cohort-II.** (a) Spearman correlations between immune indexes with Rho (R) values and *P*-values in Cohort-I. For optimal visualization, the number range 1-100 was showed in X and Y axes in some plots. (b) Comparisons of immune indexes between patients < 80y and ≥ 80y in MG and SG of Cohort-I. (c) Comparisons of $NT_{50}$ values between patients < 80y and ≥ 80y in MG and SG of Cohort-II. Wilcoxon test was used

for statistical analysis. *$P$-value ≤ 0.05; **$P$-value ≤ 0.01; ***$P$-value ≤ 0.001; ****$P$-value ≤ 0.0001; blank: $P$-value > 0.05.

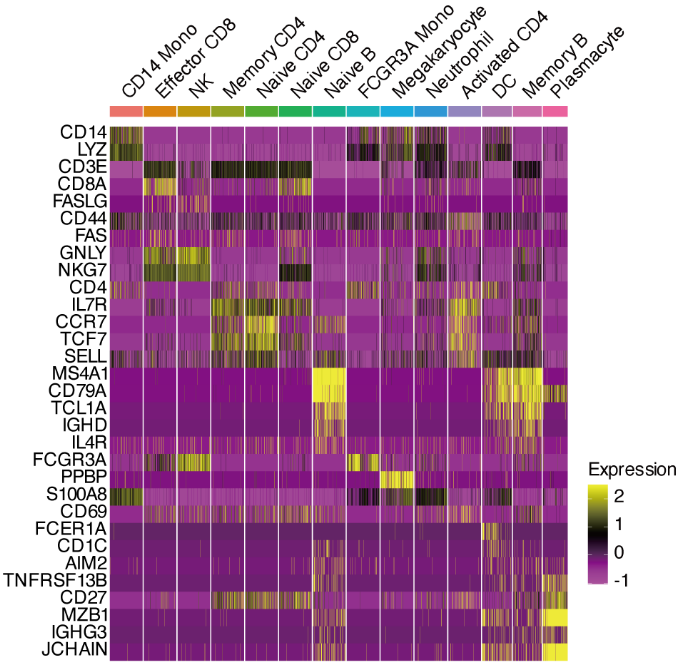


**Supplementary Fig. S2. The heatmap displaying expression of markers for cell type identification in PBMCs of Cohort-III.**

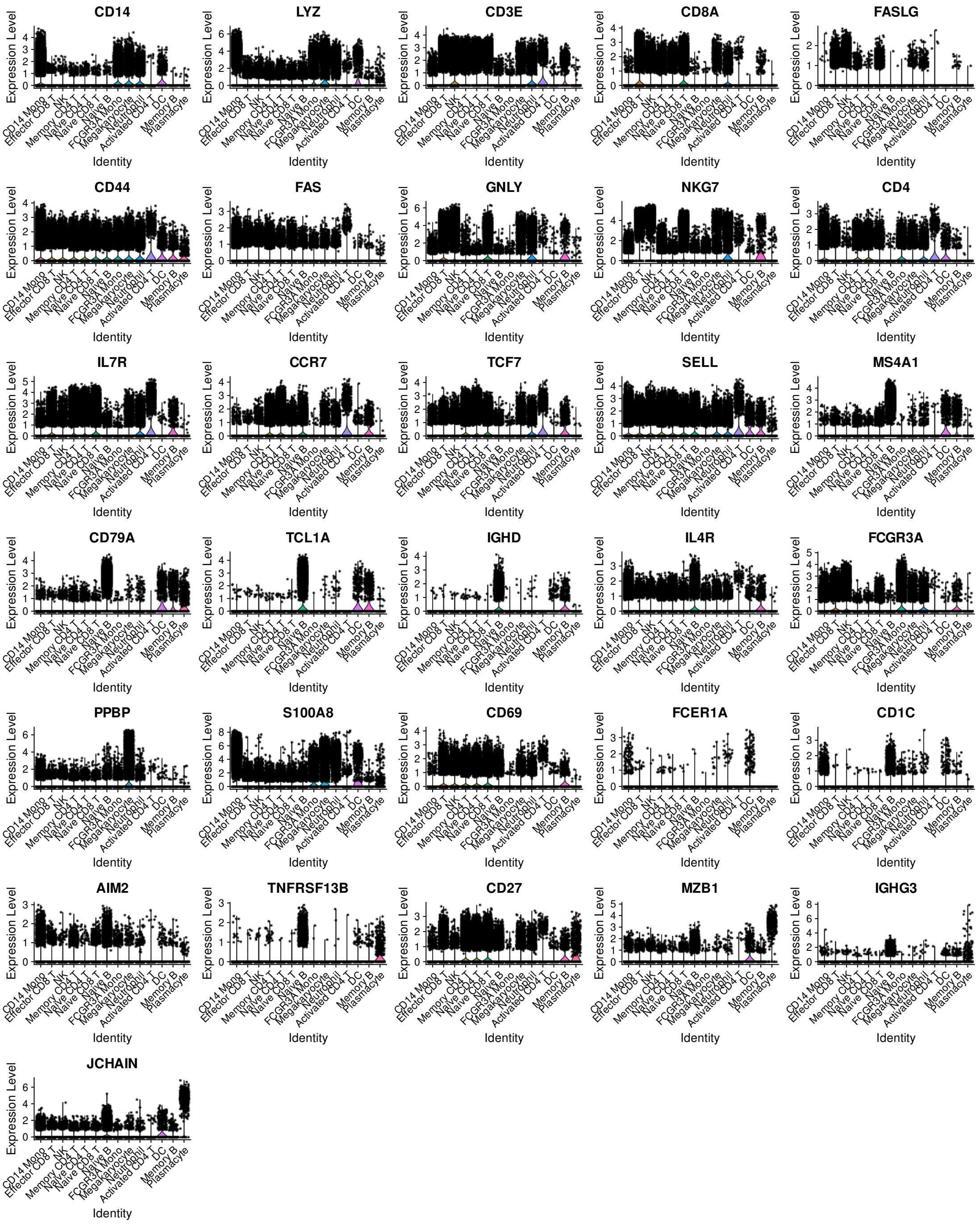


**Supplementary Fig. S3. The violin plots displaying expression of markers for cell type identification in PBMCs of Cohort-III.**

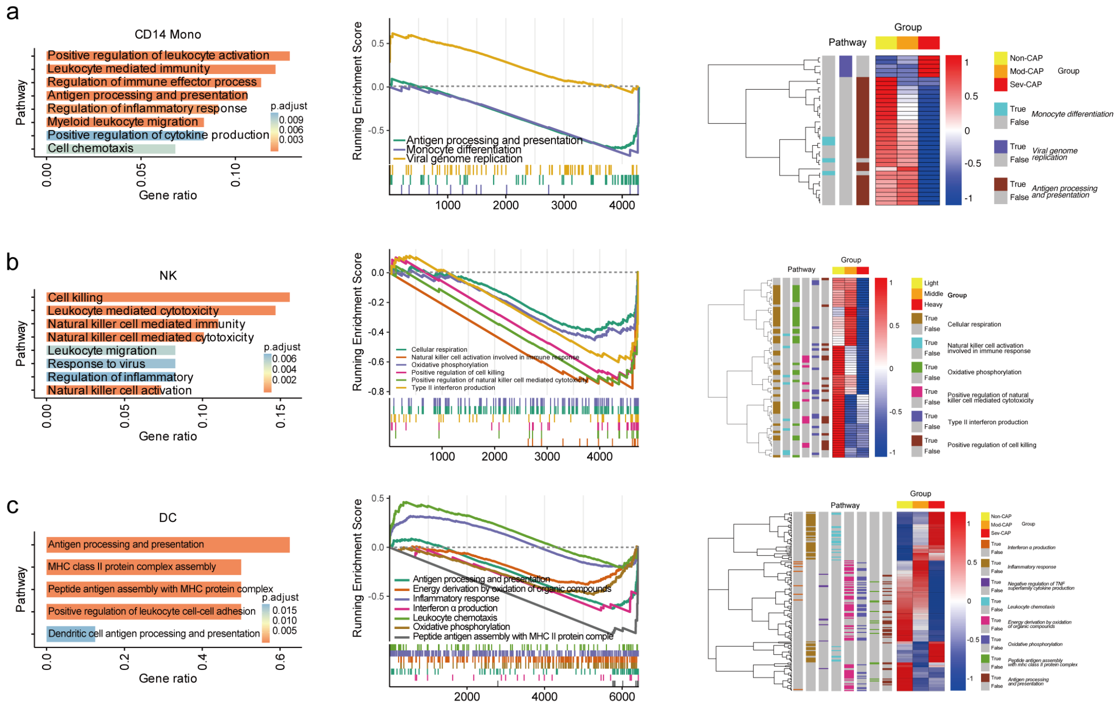


**Supplementary Fig. S4. Analysis of differential pathways and genes in innate immune cells of Cohort-III.** (a, b, c) Left: relevant GO enrichment results of common DEGs in both Mod-CAP and Sev-CAP versus Non-CAP in each cell type. X axis represented gene ratios, and column colors represented adjusted *P*-values. Middle: relevant GSEA results of Sev-CAP versus Non-CAP in each cell type. Relevant pathways were labeled and displayed as curves. X axis represented differential gene orders, and Y axis represented running enrichment scores. Vertical lines in the plot bottom represented enriched genes in these pathways. Right: heatmaps displaying expression of genes involved in these pathways in different groups. Left annotating columns indicated gene involvement in these pathways, and colors of heatmap grids represented expression. (a) CD14 Mono; (b) NK; (c) DC.

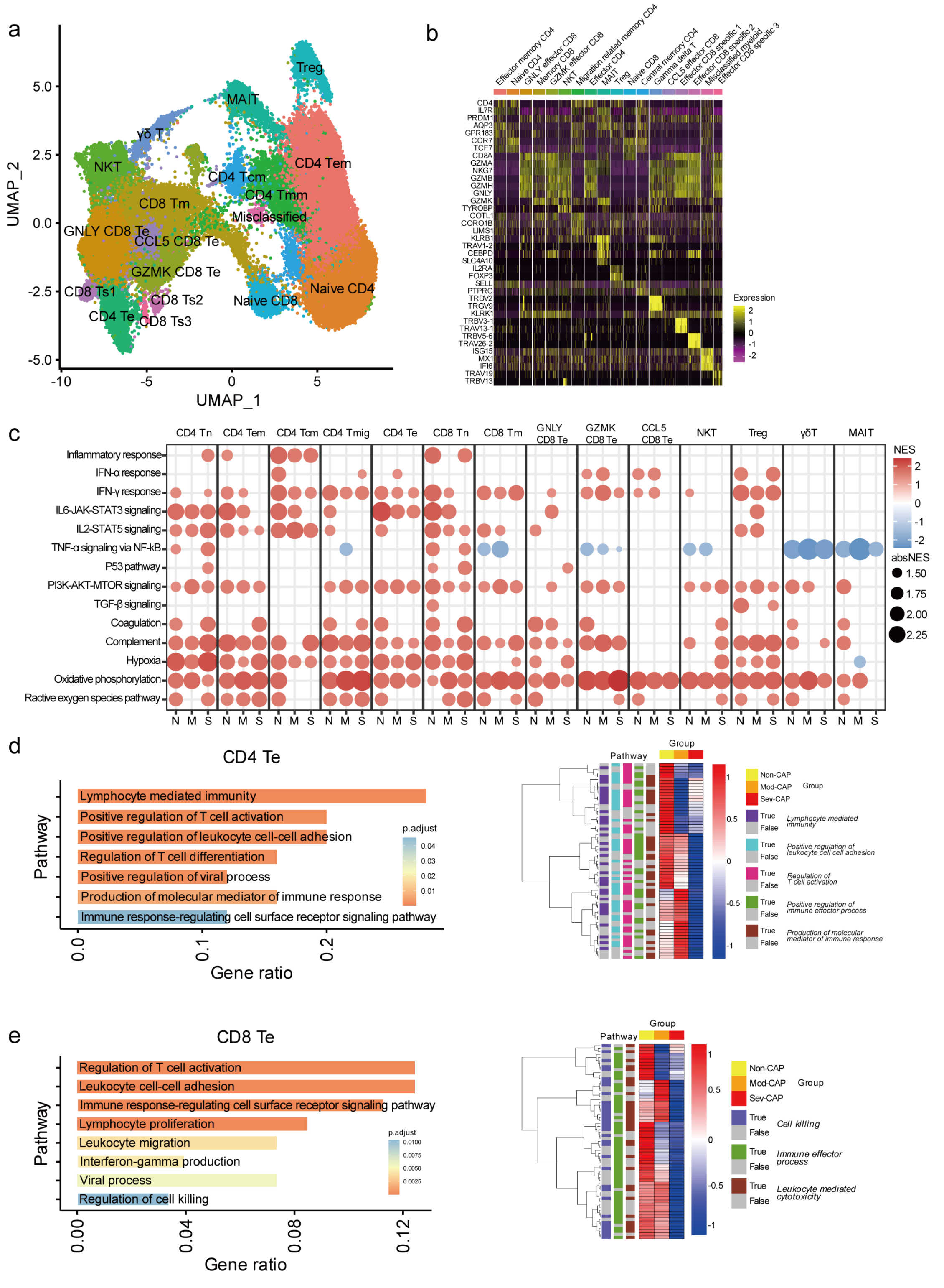


**Supplementary Fig. S5. T cell subtype identification and analysis of differential pathways and genes in T cells of Cohort-III.** (a) UMAP displaying all subtypes of T cells. Misclassified cells were excluded in further analysis. (b) The heatmap displaying

expression of cell markers for annotation of T cell subtypes. (c) GSEA results of Non-CAP, Mod-CAP, and Sev-CAP versus HRD. Dot sizes represented absolute Normalized Enrichment Scores (absNESs), and dot colors represented Normalized Enrichment Scores (NESs). Red: upregulated; blue: downregulated. (d, e) Left: relevant GO enrichment results of common DEGs in both Mod-CAP and Sev-CAP versus Non-CAP in each cell type. X axis represented gene ratios, and column colors represented adjusted *P*-values. Right: heatmaps displaying expression of genes involved in the pathways in different groups. Left annotating columns indicated gene involvement in these pathways, and colors of heatmap grids represented expression. (d) CD4 Te; (e) CD8 Te.

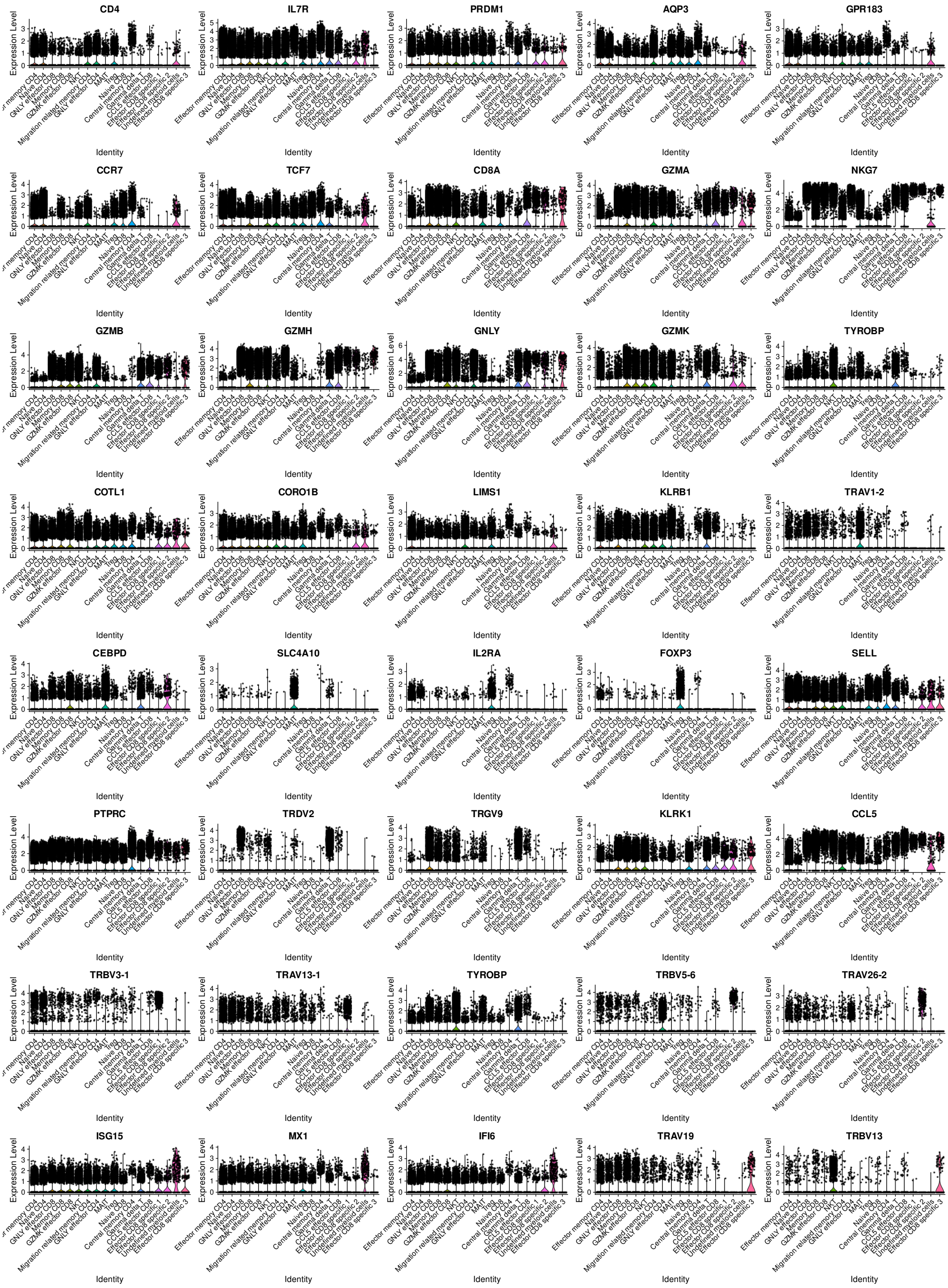


**Supplementary Fig. S6. The violin plots displaying expression of cell markers for annotation of T cell subtypes.**

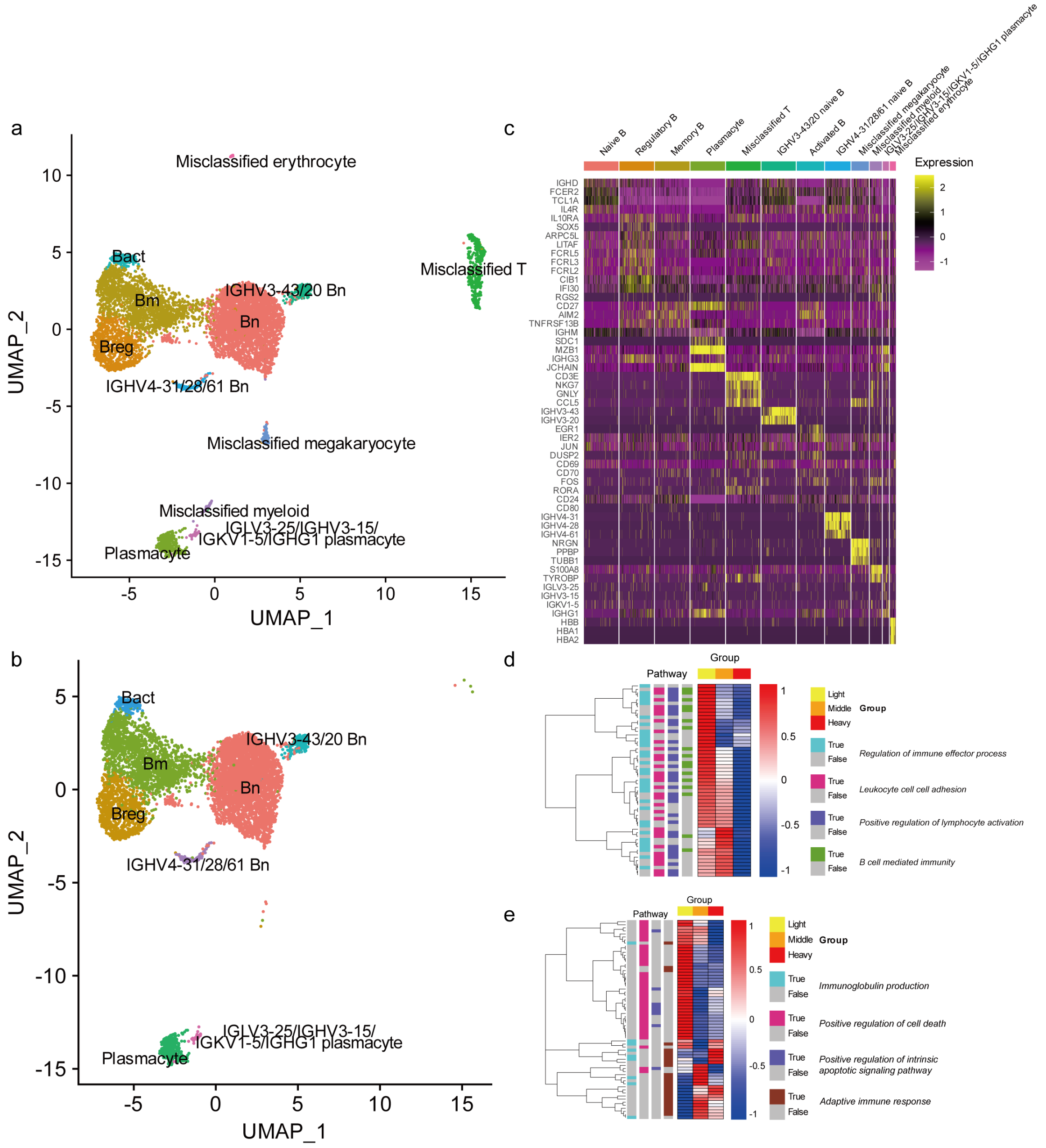


**Supplementary Fig. S7. B cell subtype identification and analysis of differential pathways and genes in B cells of Cohort-III.** (a, b) UMAP displaying all subtypes of B cells with misclassified cells (a) or without misclassified cells (b). Misclassified cells were excluded in further analysis. (c) The heatmap displaying expression of cell markers for annotation of B cell subtypes. (d, e) heatmaps displaying expression of genes involved in the pathways of GSEA results in different groups. Left annotating

columns indicated gene involvement in these pathways, and colors of heatmap grids represented expression. (d) Bm; (e) Plasmacyte.

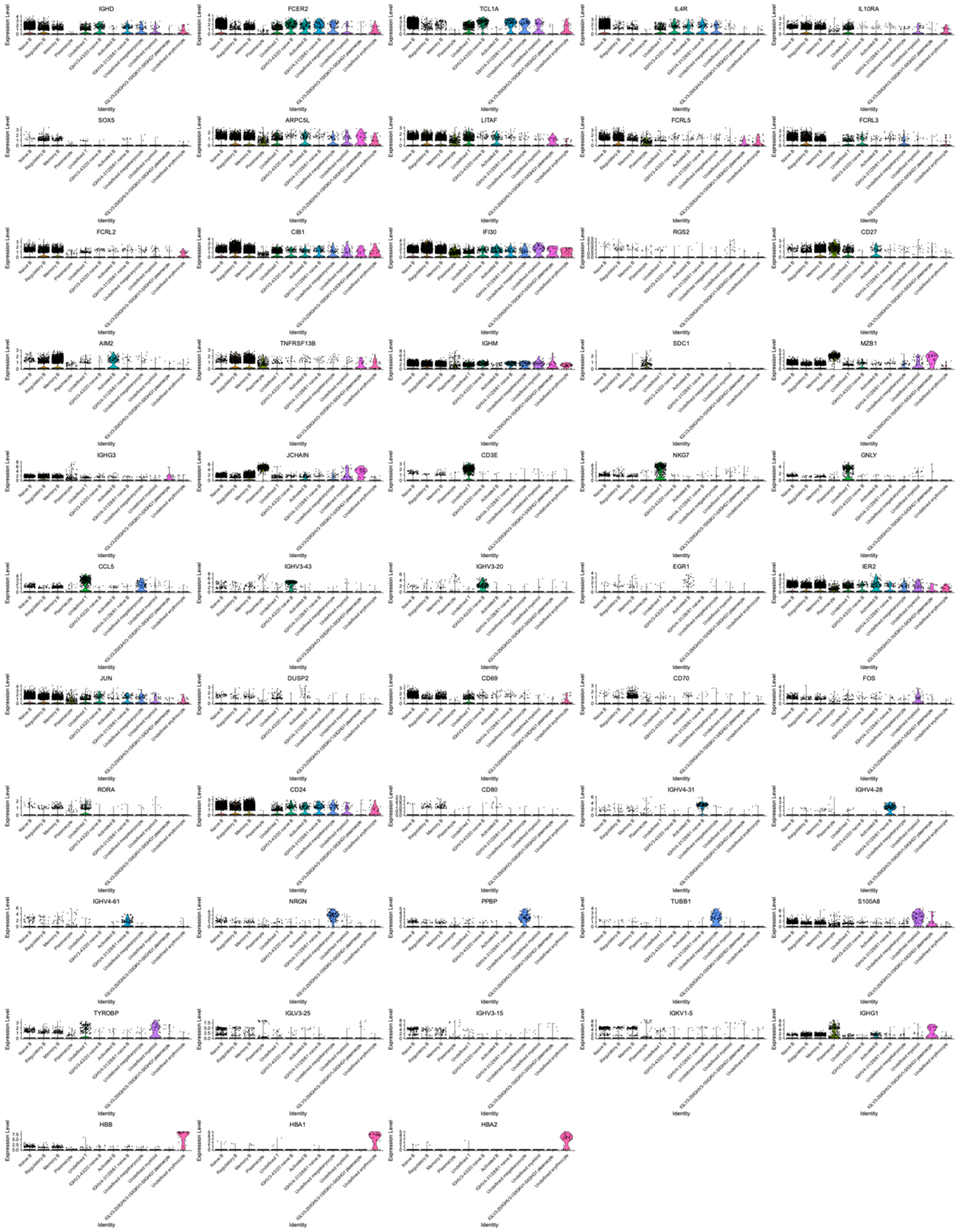


**Supplementary Fig. S8. The violin plots displaying expression of cell markers for annotation of B cell subtypes.**

## 3. Supplementary Tables

**Supplementary Table S1.** Clinical characteristics of patients in Cohort-I.

| Items | MG (n=520) | SG (n=59) | *P*-values |
|---|---|---|---|
| Age (y) | 79 [69.80, 87.40] | 86.20 [77.55, 90.35] | <0.001 |
| Gender (n) | | | |
| Male | 234 (45) | 25 (42.37) | 0.805 |
| Female | 286 (55) | 34 (57.63) | |
| Vaccination (n) | | | |
| Unvaccinated | 363 (69.81) | 43 (72.88) | 0.019 |
| Vaccinated | 56 (10.77) | 0 (0) | |
| Unknown | 101 (19.42) | 16 (27.12) | |
| Leuk ($\times 10^9$/L) | 5.14 [4.14, 6.66] | 7.08 [5.05, 9.73] | <0.001 |
| Neut ($\times 10^9$/L) | 3.28 [2.39, 4.40] | 5.09 [3.19, 8.45] | <0.001 |
| Lymp ($\times 10^9$/L) | 1.20 [0.86, 1.63] | 0.90 [0.50, 1.26] | <0.001 |
| Mono ($\times 10^9$/L) | 0.43 [0.33, 0.56] | 0.44 [0.30, 0.58] | 0.713 |
| Eosi ($\times 10^9$/L) | 0.04 [0.01, 0.10] | 0.02 [0, 0.05] | <0.001 |
| Baso($\times 10^9$/L) | 0.01 [0.01, 0.02] | 0.01 [0, 0.01] | <0.001 |
| Neut% (%) | 64.45 [56.12, 72.45] | 77.80 [69.45, 88.55] | <0.001 |
| Lymp% (%) | 24 [17.02, 32.60] | 11.50 [7.25, 18.45] | <0.001 |
| Mono% (%) | 8.30 [6.70, 10.70] | 6.40 [4.30, 8.65] | <0.001 |
| Eosi% (%) | 0.80 [0.30, 1.90] | 0.20 [0.10, 0.85] | <0.001 |
| Baso% (%) | 0.30 [0.20, 0.40] | 0.10 [0.10, 0.30] | <0.001 |
| IL-6 (pg/ml) | 28.66 [14.92, 75.88] | 48.88 [27.81, 154.95] | <0.001 |
| IL-17A (ng/ml) | 1.08 [0.34, 2.73] | 1.50 [0.49, 3] | 0.357 |
| IL-10 (ng/ml) | 4.14 [2.35, 7.21] | 8.12 [3.83, 12.48] | <0.001 |
| IFN-γ (ng/ml) | 1.72 [0.39, 5.81] | 1.27 [0.33, 5.89] | 0.645 |
| IL-2 (ng/ml) | 0.08 [0.04, 0.88] | 0.10 [0.06, 2.84] | 0.018 |
| IL-1β (ng/ml) | 0.94 [0.29, 2.03] | 0.89 [0.27, 2.50] | 0.846 |
| IL-5 (ng/ml) | 0.07 [0.03, 0.12] | 0.08 [0.04, 0.34] | 0.324 |
| IL-12 (ng/ml) | 0.07 [0.05, 0.49] | 0.07 [0.04, 0.62] | 0.746 |
| IL-8 (ng/ml) | 110.90 [34.21, 243.57] | 94.09 [39.42, 180.94] | 0.232 |
| IL-4 (ng/ml) | 0.74 [0.07, 3.19] | 0.68 [0.08, 1.65] | 0.696 |
| TNF-α (ng/ml) | 6.57 [2.90, 12.34] | 3.25 [1.02, 7.14] | <0.001 |
| Immunoglobulin (g/L) | 22.32 [19.75, 25.06] | 23.89 [21.52, 26.52] | 0.006 |

Note: 1. Abbreviations of items used in this table: Leuk (leukocyte count), Neut (neutrophil count), Lymp (lymphocyte count), Mono (monocyte count), Eosi

(eosinophil count), Baso (basophil count), Neut% (neutrophil percentage), Lymp% (lymphocyte %), Mono% (monocyte percentage), Eosi% (eosinophil percentage), and Baso% (basophil percentage). 2. Normally distributed continuous variables were displayed as Mean (SD), nonnormally distributed continuous variables were displayed as Medium [25th, 75th], and categorical variables were displayed as number (%). 3. Unpaired t-test was used for statistical analysis of normally distributed continuous variables, Wilcoxon signed-rank test was used for statistical analysis of nonnormally distributed continuous variables, and Pearson's chi-squared test was used for statistical analysis of categorical variables.

**Supplementary Table S2.** Clinical characteristics of patients in Cohort-II.

| Items | MG (n=10) | SG (n=10) | *P*-value |
|---|---|---|---|
| Age (y) | 85 [78.75, 87.75] | 86 [82.75, 89.25] | 0.448 |
| Gender (n) | | | |
| Female | 5 (50) | 5 (50) | 1 |
| Male | 5 (50) | 5 (50) | |
| Vaccination (n) | | | |
| Unvaccinated | 10 (100) | 10 (100) | |
| Vaccinated | 0 (0) | 0 (0) | |
| Unknown | 0 (0) | 0 (0) | |
| The $NT_{50}$ values of WT-D614G | 39.90 [9.52, 922.25] | 0.10 [0.10, 23.60] | 0.007 |
| The $NT_{50}$ values of BA.1 | 398.10 [183.32, 448.80] | 92.39 [50.53, 134] | 0.01 |
| The $NT_{50}$ values of BA.2 | 3710.50 [2800.25, 4586] | 542.75 [131.93, 1088.50] | 0.008 |
| The $NT_{50}$ values of BA.5.2 | 489.45 [302.17, 1086.30] | 73.60 [33.92, 143.22] | 0.001 |
| The $NT_{50}$ values of BQ.1 | 79.42 [49.53, 154.35] | 24.19 [5, 96.92] | 0.226 |
| The $NT_{50}$ values of XBB.1.5 | 29.59 [13.42, 106.24] | 5.80 [2, 23.40] | 0.041 |

Note: 1. Normally distributed continuous variables were displayed as Mean (SD), nonnormally distributed continuous variables were displayed as Medium [25th, 75th], and categorical variables were displayed as number (%). 2. Unpaired *t*-test was used for statistical analysis of normally distributed continuous variables, Wilcoxon signed-rank test was used for statistical analysis of nonnormally distributed continuous variables, and Fisher 's exact probability test was used for statistical analysis of categorical variables.

**Supplementary Table S3.** Clinical characteristics of patients in Cohort-III.

| Items | HRD (n=3) | Non-lvCAP (n=3) | Mod-lvCAP (n=3) | Sev-lvCAP (n=7) | *P*-value |
|---|---|---|---|---|---|
| Age (y) | 67.00 (4.58) | 73.33 (9.29) | 64.33 (12.22) | 77.00 (9.83) | 0.246 |
| Gender (n) | | | | | |
| Female | 1 (33.33) | 1 (33.33) | 2 (66.67) | 2 (28.57) | 0.882 |
| Male | 2 (66.67) | 2 (66.67) | 1 (33.33) | 5 (71.43) | |
| Vaccination (n) | | | | | |
| Unvaccinated | 3 (100.00) | 3 (100.00) | 3 (100.00) | 7 (100.00) | |
| Vaccinated | 0 (0.00) | 0 (0.00) | 0 (0.00) | 0 (0.00) | |
| Unknown | 0 (0.00) | 0 (0.00) | 0 (0.00) | 0 (0.00) | |
| ULD (n) | 2.00 [1.50, 2.00] | 2.00 [2.00, 2.50] | 2.00 [1.50, 4.00] | 3.00 [2.50, 3.50] | 0.417 |
| ORF1ab Ct | - | 28.00 (3.86) | 30.21 (1.02) | 28.22 (3.66) | 0.658 |
| N Ct | - | 28.34 (3.74) | 30.45 (1.93) | 28.15 (3.99) | 0.656 |
| Neut% (%) | 59.43 (10.70) | 69.03 (7.32) | 67.00 (8.25) | 81.34 (11.38) | 0.04 |
| Neut ($\times 10^9$/L) | 3.50 [3.26, 3.89] | 5.62 [4.44, 6.00] | 3.08 [3.02, 3.68] | 6.96 [5.31, 11.86] | 0.049 |
| MPV (fL) | 9.50 [9.45, 10.10] | 10.80 [10.15, 10.90] | 9.80 [9.55, 10.15] | 10.90 [9.50, 12.55] | 0.605 |
| Mono% (%) | 5.93 (0.96) | 6.40 (1.08) | 6.20 (3.36) | 6.44 (4.02) | 0.996 |
| Mono ($\times 10^9$/L) | 0.36 (0.09) | 0.45 (0.07) | 0.31 (0.15) | 0.57 (0.34) | 0.436 |
| MCV (fL) | 90.53 (5.23) | 88.97 (0.55) | 92.53 (7.25) | 92.44 (4.74) | 0.738 |
| PLT ($\times 10^9$/L) | 254.33 (50.20) | 241.00 (21.70) | 212.33 (93.07) | 216.43 (87.93) | 0.864 |
| RBC ($\times 10^{12}$/L) | 4.27 (0.02) | 4.26 (0.38) | 3.83 (0.28) | 4.01 (0.74) | 0.718 |
| RSD (fL) | 44.10 (3.58) | 42.00 (3.56) | 46.53 (2.76) | 45.94 (3.82) | 0.384 |
| RCV (%) | 13.37 (1.78) | 13.07 (0.90) | 13.73 (0.35) | 13.49 (0.75) | 0.863 |
| CRP (mg/L) | 0.80 [0.50, 0.85] | 9.09 [6.39, 16.67] | 11.53 [6.38, 18.73] | 27.24 [11.16, 60.73] | 0.041 |
| MCHC (g/L) | 333.00 [328.00, 341.00] | 328.00 [327.00, 331.00] | 327.00 [327.00, 329.50] | 331.00 [329.50, 333.50] | 0.645 |
| Leuk ($\times 10^9$/L) | 5.97 [5.75, 6.35] | 8.25 [6.68, 8.39] | 4.92 [4.38, 5.92] | 9.45 [6.82, 13.19] | 0.152 |
| Baso ($\times 10^9$/L) | 0.03 [0.03, 0.04] | 0.04 [0.03, 0.04] | 0.01 [0.00, 0.01] | 0.00 [0.00, 0.01] | 0.064 |
| Baso% (%) | 0.47 (0.15) | 0.47 (0.12) | 0.20 (0.10) | 0.10 (0.15) | 0.004 |
| Eosi ($\times 10^9$/L) | 0.14 (0.03) | 0.22 (0.08) | 0.10 (0.11) | 0.10 (0.08) | 0.178 |
| Eosi% (%) | 2.30 (0.46) | 3.40 (2.19) | 1.93 (2.22) | 1.21 (0.99) | 0.236 |
| HCT (%) | 39.20 [37.75, 39.80] | 36.60 [36.10, 39.10] | 35.30 [35.20, 35.35] | 39.00 [34.70, 40.65] | 0.308 |

| | | | | | |
|---|---|---|---|---|---|
| HGB (g/L) | 129.67 (10.26) | 125.00 (12.29) | 116.00 (1.73) | 122.71 (17.44) | 0.69 |
| Lymp ($\times 10^9$/L) | 1.94 (0.69) | 1.50 (0.61) | 1.38 (0.89) | 0.90 (0.46) | 0.14 |
| Lymp% (%) | 31.87 (10.12) | 20.70 (6.23) | 24.67 (8.91) | 10.90 (7.11) | 0.012 |
| MCH (pg) | 29.70 [28.95, 31.40] | 29.20 [29.15, 29.40] | 30.20 [29.05, 31.70] | 30.20 [29.35, 32.35] | 0.763 |
| LPR (%) | 23.10 [22.90, 27.20] | 31.60 [27.45, 32.50] | 25.20 [23.05, 27.70] | 31.70 [22.90, 43.90] | 0.573 |
| LPC ($\times 10^9$/L) | 64.00 (4.58) | 71.33 (15.53) | 53.33 (22.30) | 65.00 (13.03) | 0.516 |
| PWD | 16.20 [16.15, 16.30] | 16.40 [16.30, 16.40] | 16.30 [16.15, 16.35] | 16.30 [15.95, 16.75] | 0.879 |
| PV (%) | 0.19 (0.13) | 0.25 (0.04) | 0.21 (0.09) | 0.23 (0.05) | 0.742 |
| AST (U/L) | - | 17.45 [13.06, 24.62] | 34.54 [27.25, 35.84] | 42.28 [27.38, 47.86] | 0.208 |
| ALT (U/L) | - | 21.88 [18.25, 33.16] | 19.71 [15.64, 62.30] | 33.25 [29.56, 53.09] | 0.353 |
| AST/ALT | - | 0.72 [0.66, 0.76] | 1.73 [1.03, 1.80] | 0.76 [0.53, 1.81] | 0.942 |
| ALP (U/L) | - | 70.26 [66.80, 83.46] | 76.47 [73.12, 79.34] | 76.69 [58.46, 89.42] | 0.939 |
| ALB (g/L) | - | 38.61 (3.75) | 37.55 (1.19) | 35.27 (4.34) | 0.421 |
| A/G | - | 1.11 (0.22) | 1.27 (0.33) | 1.24 (0.12) | 0.579 |
| TP (g/L) | - | 73.85 (5.68) | 68.50 (7.12) | 63.73 (7.18) | 0.147 |
| TB (μmol/L) | - | 10.93 (3.03) | 8.60 (2.93) | 8.59 (2.58) | 0.462 |
| Na (mmol/L) | - | 142.00 [141.00, 143.00] | 142.00 [141.00, 144.00] | 139.00 [138.50, 143.00] | 0.506 |
| K (mmol/L) | - | 3.62 [3.54, 3.77] | 3.67 [3.55, 3.84] | 3.93 [3.67, 4.66] | 0.577 |
| GGT (U/L) | - | 37.22 [27.67, 39.04] | 32.53 [25.41, 126.29] | 38.01 [26.62, 50.24] | 0.778 |
| eGFR | - | 78.67 (15.63) | 94.33 (31.18) | 111.71 (71.67) | 0.704 |
| Crea (μmol/L) | - | 76.70 [70.40, 93.05] | 51.90 [51.30, 82.90] | 55.60 [37.80, 113.20] | 0.592 |
| Cl (mmol/L) | - | 104.00 [103.50, 104.50] | 102.00 [101.50, 104.00] | 102.00 [98.50, 104.50] | 0.62 |

Note: 1. Multiple indexes were compared among groups to diminish heterogeneity among patients. 2. Abbreviations of items used in this table: ULN (numbers of underlying diseases), ORF1ab Ct (SARS-CoV-2 ORF1ab gene threshold cycle), N Ct

(SARS-CoV-2 N gene threshold cycle), Neut% (neutrophil percentage), Neut (neutrophil count), MPV (mean platelet volume), Mono% (monocyte percentage), Mono (monocyte count), MCV (mean corpuscular volume), PLT (platelet count), RBC (red blood cell count), RSD (red blood cell distribution width SD), RCV (red blood cell distribution width CV), CRP (C-reactive protein), MCHC (mean hemoglobin concentration), Leuk (leukocyte count), Baso (basophil count), Baso% (basophil percentage), Eosi (eosinophil count), Eosi% (eosinophil percentage), HCT (hematocrit), HGB (hemoglobin), Lymp (lymphocyte count), Lymp% (lymphocyte %), MCH (mean hemoglobin), LPR (large platelet ratio), LPC (large platelet count), PWD (platelet distribution width), PV (platelet volume), AST (aspartate aminotransferase), ALT (alanine aminotransferase), ALP (alkaline phosphatase), ALB (albumin), A/G (albumin /globulin), TP (total protein), TB (total bilirubin), Na (sodium), K (Potassium), γ-GGT (γ-glutamyl transferase), eGFR (estimated glomerular filtration rate), Crea (creatinine), and Cl (chlorine). 3. Normally distributed continuous variables were displayed as Mean (SD), nonnormally distributed continuous variables were displayed as Medium [$25^{th}$, $75^{th}$], and categorical variables were displayed as number (%). 4. One-way ANOVA was used for statistical analysis of normally distributed continuous variables, Kruskal-Wallis H test was used for statistical analysis of nonnormally distributed continuous variables, and Fisher ’s exact probability test was used for statistical analysis of categorical variables. 5. “-” in the table represented missing values.

**Supplementary Table S4. Cell types and subtypes with identification markers.**

| Cell resource | Cell type | Abbreviation | Cell markers |
|---|---|---|---|
| PBMCs | $CD14^+$ monocyte | CD14 Mono | CD14; LYZ |
| | Effector CD8 T cell | Effector CD8 T | CD3E; CD8A; FASLG; CD44; FAS |
| | Natural killer cell | NK | GNLY; NKG7 |
| | Memory CD4 T cell | Memory CD4 T | CD3E; CD4; IL7R |
| | Naïve CD4 T cell | Naïve CD4 T | CD3E; CD4; CCR7; TCF7 |
| | Naïve CD8 T cell | Naïve CD8 T | CD3E; CD8A; CCR7; SELL |
| | Naïve B cell | Naïve B | MS4A1; CD79A; TCL1A; IGHD; IL4R |
| | $FCGR3A^+$ monocyte | FCGR3A Mono | FCGR3A; LYZ |
| | Megakaryocyte | Megakaryocyte | PPBP |
| | Neutrophil | Neutrophil | S100A8 |
| | Activated CD4 T cell | Activated CD4 T | CD3E; CD4; CD69 |
| | Dendritic cell | DC | FCER1A; CD1C |
| | Memory B cell | Memory B | MS4A1; CD79A; AIM2; TNFRSF13B; CD27 |
| | Plasmacyte | Plasmacyte | MS4A1; CD79A; MZB1; IGHG3; JCHAIN |
| T cells | Naïve CD4 T cell | CD4 Tn | CD4; CCR7; TCF7 |
| | Effector memory CD4 T cell | CD4 Tem | CD4; IL7R; PRDM1; AQP3; GPR183 |
| | Central memory CD4 T cell | CD4 Tcm | CD4; CCR7; SELL; IL7R; PTPRC |
| | Migration-related memory CD4 T cell | CD4 Tmm | CD4; IL7R; COTL1; CORO1B; LIMS1 |
| | $GNLY^+$ effector CD4 T cell | CD4 Te | CD4; NKG7; GNLY; GZMA; GZMH |
| | Naïve CD8 T cell | CD8 Tn | CCR7; SELL |
| | Memory CD8 T cell | CD8 Tm | CD8A; IL7R; AQP3; GZMK |
| | $GZMK^+$ effector CD8 T cell | GZMK CD8 Te | CD8A; GZMA; NKG7; GZMK |

| | | | |
|---|---|---|---|
| | GNLY$^{+}$ effector CD8 T cell | GNLY CD8 Te | CD8A; GZMA; NKG7; GZMB; GZMH; GNLY |
| | CCL5$^{+}$ effector CD8 T cell | CCL5 CD8 Te | CD8A; GZMA; NKG7; GZMK; CCL5 |
| | Natural killing T cell | NKT | CD8A; TYROBP |
| | Mucosal-associated invariant T cell | MAIT | KLRB1; TRAV1-2; CEBPD; SLC4A10 |
| | Gamma-delta T cell | γδT | TRDV2; TRGV9; KLRK1; TRGV9 |
| | Regulatory T cell | Treg | IL2RA; FOXP3 |
| | Effector CD8 T cell specific cluster 1 | CD8 Ts1 | CD8A; GZMA; NKG7; TRBV3-1; TRAV13-1 |
| | Effector CD8 T cell specific cluster 2 | CD8 Ts2 | CD8A; GZMA; NKG7; TRBV5-6; TRAV26-2 |
| | Effector CD8 T cell specific cluster 3 | CD8 Ts3 | CD8A; GZMA; NKG7; TRAV19; TRBV13 |
| | Misclassified myeloid cell | Misclassified | ISG15; MX1; IFI6 |
| B cells | Naive B cell | Bn | IGHD; FCER2; TCL1A; IL4R |
| | Regulatory B cell | Breg | IL10RA; SOX5; ARPC5L; LITAF; FCRL5; FCRL3; FCRL2; CIB1; IFI30; RGS2 |
| | Memory B cell | Bm | CD27; AIM2; TNFRSF13B; IGHM; IGHD |
| | Activated B cell | Bact | EGR1; IER2; JUN; DUSP2; CD69; CD70; FOS; RORA; CD24; CD80 |
| | Plasmacyte | Plasmacyte | SDC1; MZB1; IGHG3; JCHAIN |
| | IGHV3-43/20$^{high}$ naive B cell | IGHV3-43/20 Bn | IGHV3-43; IGHV3-20; IGHD; FCER2; TCL1A; IL4R |
| | IGHV4-31/28/61$^{high}$ naive B | IGHV4-31/28/61 Bn | IGHV4-31; IGHV4-28; IGHV4-61; IGHD; FCER2; TCL1A; IL4R |
| | IGLV3-25/IGHV3-15/IGKV1-5/IGHG1$^{high}$ plasmacyte | IGLV3-25/IGHV3-15/IGKV1-5/IGHG1 plasmacyte | SDC1; MZB1; IGHG3; JCHAIN; IGLV3-25; IGHV3-15; IGKV1-5; IGHG1 |
| | Misclassified megakaryocyte | Misclassified megakaryocyte | NRGN; PPBP; TUBB1 |

| | | | |
|---|---|---|---|
| | Misclassified myeloid cell | Misclassified myeloid | S100A8; TYROBP |
| | Misclassified T | Misclassified T | CD3E; NKG7; GNLY; CCL5 |
| | Misclassified erythrocyte | Misclassified erythrocyte | HBB; HBA1; HBA2 |

Note: 1. CD4 Tmm was a cluster of CD4 Tm highly expressing migration-related genes (COTL1, CORO1B, and LIMS1). 2. CD8 Ts1/2/3 were clusters of effector CD8 T cells mainly coming from some patients (CD8 Ts1: Non-CAP_2; CD8 Ts2: Mod-CAP_1; CD8 Ts3: Non-CAP_1) with high expression of specific TCR genes (listed Supplementary Table S4). 3. IGLV3-25/IGHV3-15/IGKV1-5/IGHG1 plasmacyte was a little cluster of plasmacytes isolated from the major plasmacyte cluster and highly expressing immunoglobulin genes IGLV3-25, IGHV3-15, IGKV1-5, and IGHG1.

**Supplementary Table S5.** Gene list of pathways used for AddModuleScore_UCell.

| Pathway | Genes |
| --- | --- |
| Antigen processing and presentation [7] | KEGG_ANTIGEN_PROCESSING_AND_PRESENTATION (KEGG pathway: hsa04612) |
| FcγR-mediated phagocytosis [7] | KEGG_FC_GAMMA_R_MEDIATED_PHAGOCYTOSIS (KEGG pathway: hsa04666) |
| Chemokine signal pathway [7] | KEGG_CHEMOKINE_SIGNALING_PATHWAY (KEGG pathway: hsa04062) |
| Cell killing and cytotoxicity [7] | KEGG_NATURAL_KILLER_CELL_MEDIATED_CYTOTOXICITY (KEGG pathway: hsa04650) |
| Activation score (T cell) [8] | CD69, HLA-DRB1, HLA-DRA, HLA-DRB3, HLA-DRB4, HLA-DRB5, IL2RA, CD38, MKI67 |
| Cytotoxicity score (T cell) [9] | RF1, IFNG, GNLY, NKG7, GZMB, GZMA, GZMH, KLRK1, KLRB1, KLRD1, CTSW, CST7 |
| Th1 score [10] | IFNG, IL2, TBX21, CXCR3, DPP4, CD27 |
| MATI function [10] | KLRB1, TRAV1-2, CEBPD, SLC4A10 |
| γδT function [10] | TRGV9, TRDC, TRDV2, TRGC1 |

Note: Genes used in these pathways referred to References.

## 4. References


1 Xia, S. *et al.* SARS-CoV-2 Omicron subvariants exhibit distinct fusogenicity, but similar sensitivity, to pan-CoV fusion inhibitors. *Emerg Microbes Infect* **12**, 2178241, doi:10.1080/22221751.2023.2178241 (2023).

2 Hao, Y. *et al.* Integrated analysis of multimodal single-cell data. *Cell* **184**, 3573-3587 e3529, doi:10.1016/j.cell.2021.04.048 (2021).

3 Wu, T. *et al.* clusterProfiler 4.0: A universal enrichment tool for interpreting omics data. *Innovation (Camb)* **2**, 100141, doi:10.1016/j.xinn.2021.100141 (2021).

4 Subramanian, A. *et al.* Gene set enrichment analysis: a knowledge-based approach for interpreting genome-wide expression profiles. *Proc Natl Acad Sci U S A* **102**, 15545-15550, doi:10.1073/pnas.0506580102 (2005).

5 ImmunoMind Team Immunarch: An R Package for Painless Bioinformatics Analysis of T-Cell and B-Cell Immune Repertoires. *Zenodo* **10**, doi:10.5281/zenodo.3367200 (2019).

6 Koch, H., Starenki, D., Cooper, S. J., Myers, R. M. & Li, Q. powerTCR: A model-based approach to comparative analysis of the clone size distribution of the T cell receptor repertoire. *PLoS Comput Biol* **14**, e1006571, doi:10.1371/journal.pcbi.1006571 (2018).

7 *KEGG PATHWAY Database*, <https://www.genome.jp/kegg/pathway.html> (2023).

8 Rea, I. M., McNerlan, S. E. & Alexander, H. D. CD69, CD25, and HLA-DR activation antigen expression on CD3+ lymphocytes and relationship to serum TNF-alpha, IFN-gamma, and sIL-2R levels in aging. *Exp Gerontol* **34**, 79-93, doi:10.1016/s0531-5565(98)00058-8 (1999).

9 Zhang, J. Y. *et al.* Single-cell landscape of immunological responses in patients with COVID-19. *Nat Immunol* **21**, 1107-1118, doi:10.1038/s41590-020-0762-x (2020).

10 Hu, C. *et al.* CellMarker 2.0: an updated database of manually curated cell markers in human/mouse and web tools based on scRNA-seq data. *Nucleic Acids Res* **51**, D870-D876, doi:10.1093/nar/gkac947 (2023).
